# Multi-Criteria Decision Making to Detect Multiple Moving Targets in Radar Using Digital Codes

**DOI:** 10.3390/s22093176

**Published:** 2022-04-21

**Authors:** Majid Alotaibi

**Affiliations:** Department of Computer Engineering, College of Computer and Information Systems, Umm Al-Qura University, Makkah 24382, Saudi Arabia; mmgethami@uqu.edu.sa

**Keywords:** Doppler radar, target detection, digital codes, windows, mat-lab

## Abstract

Technological advancement in battlefield and surveillance applications switch the radar investigators to put more effort into it, numerous theories and models have been proposed to improve the process of target detection in Doppler tolerant radar. However, still, more effort is needed towards the minimization of the noise below the radar threshold limit to accurately detect the target. In this paper, a digital coding technique is being discussed to mitigate the noise and to create clear windows for desired target detection. Moreover, multi-criteria of digital code combinations are developed using discrete mathematics and all designed codes have been tested to investigate various target detection properties such as the auto-correlation, cross-correlation properties, and ambiguity function using mat-lab to optimize and enhance the static and moving target in presence of the Doppler in a multi-target environment.

## 1. Introduction

To keep an eye on objects (static or moving), a radar system is an alone equipment to investigate the characteristics of the object for position and velocity. The radar system broadcasts electromagnetic waves in the direction of the target and takes the wave echo by the target to observe the different parameters such as range, the velocity of the desired target. The present-day radar system exploits many aerial fundamentals to transmit and receive the echoes to increase the probability of target detection. In the addition phase, array radars broadcast entirely consistent waveforms (probably scaled with the help of a complex constant). These waveforms are produced from their ‘M’ dissimilar broadcast antenna fundamentals to form a powerful transmitted signal in the favored path. Formation of the beam is performed merely by the receiving antenna array to guess the parameters of the radar to estimate the current position of the target. Therefore the broadcast degrees of freedom are restricted to one and the receiver’s degrees of freedom are more than one (simply we can say ‘n’). But multiple input and multiple output radars transmit unstable signals, these signal waveforms are obtained from their dissimilar broadcasting aerial fundamentals and use combined processing of the acknowledged signals from the dissimilar receiver array fundamentals. Though phase array radars make use of the only spatial variety, multiple input and multiple output radars utilize together spatial and signal waveform variety to get better results of the performance of the system. MIMO radars can be extensively spaced or collocated antennas [1,2]. Whereas the former design enhances the selection of the target detection to get better results. This pattern of target detection improves the resolution of the target and intervention refusal ability. However, the signal waveforms for MIMO radars affect the range. Whereas the signal waveform for single input single output (SISO) radar is considered for desired Doppler and delay resolution properties. The signal waveforms selected for MIMO radars be supposed to have desirable uncertainty in the delay. The resolution characteristics of the broadcast signal waveforms are considered with the help of the uncertainty function [3]. The uncertainty function of a broadcast signal waveform presents the matched filter output in the existence of Doppler and delay variance. If the delay (90 ms) and Doppler resolution are high (80–100 kHz), the uncertainty function is supposed to resemble a “thumbtack”. The idea of the uncertainty function is to complete the MIMO system proposed by Antonio and Robey [4]. Chun and Vaidyanathan [5] have proved the characteristics of multiple input uncertainties function and further extended the MIMO uncertainty function for frequency hopping signal waveforms. The Phase coded pulse waveforms are frequently used for radar applications because they are having a high bandwidth-time product (500–100). Numerous phase-coded signal waveforms are presented formerly [6,7,8,9] having better cross-correlation and autocorrelation properties. Such signal waveforms are intended for high-quality Doppler and delay resolution properties. On the other hand, manipulating the phase-coded signal waveforms by optimizing the MIMO uncertainty function modifies the Doppler, delay and also improves the spatial resolution characteristics.

The existing approaches in radar employ optimization algorithms with a huge number of iterations which may increase the delay, to obtain the codes with good auto or cross-correlation and mitigate the noise in the detection of static targets. Doppler tolerant digital codes are also implemented by the state of the art researchers to detect the moving targets. But these codes may not have good auto and cross-correlation properties. In this paper, an approach is presented in which various multi-criteria of digital codes have been generated with optimal auto and cross-correlation properties to mitigate the noise and the same code is tested for Doppler tolerance to detect static and multi moving targets by creating a huge number of clear windows within the threshold limit of radar detection. Each of the codes performs better for all three parameters; it is the optimization of all the three functions to efficiently detect the desired object. The binary sequences with different bit lengths in this approach are generated by using discrete mathematical operations to the digital sequence.

The rest of the paper is organized as follows. In Section 2 we present the related work, Section 3 presents our proposed approach, in Section 4 discussion about the proposed approach is presented and the paper is concluded in Section 5.

## 2. Related Work

Lewis and Kretschmer [10,11] presented two different approaches in which they recommended extra codes called P3 and P4 codes and are created from LFMWT. Moreover, these codes are used to improve the Doppler tolerance, in particular when compared to P1 and P2 codes. In addition peak side lobes of P3 and P4 codes are enhanced further to overcome the gaps that existed in polyphase codes. But the performance of these codes corrupts a smaller amount with an increase in Doppler frequency. Lewis [12] proposed a method based on windowing mode to diminish the range versus time effects of side lobes in polyphase codes considerably. However, this technique simply decreases the peak sidelobe and an ingredient amount irrespective of the successful pulse compression ratio. Kretschmer and Welch [13] projected a method in which they demonstrated that the autocorrelation function of polyphase codes is having undesired range side lobes. Hence cannot be suitable to detect multi-target detection. This approach also discussed the effect of Sidelobe reduction with the help of amplitude weighting function (AWF) of polyphase codes in the receiver filter. Even though weighed windows whenever used in source and destination (i.e., Transmitter and Receiver), provides improved results. In this approach author also proved that the weight on the sender (Receiver) is more ideal as the weight on destination (transmit) results in power loss because the existing source (transmitter) power cannot be entirely utilized. Luszczyk and Mucha [14] proposed a model to reduce the effect of the range side lobe. In this approach, they used the Kaiser-Bessel weighing function of the p4 pulse compression signal waveform to reduce the range side lobes of P4. However, whenever a high Doppler occurs, the performance of this approach shows a poor response. Ajit Kumar Sahoo and Ganapati Panda [15] anticipated a firmness windowing method to minimize the consequences of side lobes in Doppler tolerant radars. However, the presented technique experiences a delay and cannot produce bigger windows or enhance the count of windows to sense numerous moving objects correctly. Sharma and Rajeswari Rajeswari [16] proposed a model to represent optimization for multiple input multiple output radar ambiguities. However due to huge mathematical complexity delay increases, also this approach has not had a large window so the probability of missing the target is more. Reddy and Anuradha [17] presented an approach, to improve the Signal to noise ratio of Mesosphere- Stratosphere-Troposphere (MST) with the help of Kaiser hamming and cosh hamming window function. Also, this approach increases the energy of the main lobe to amplify the merit factor; however, this approach fails to remove the effect of side lobes. Because the Doppler change continuously. Therefore cannot be suitable to detect multiple moving targets. Lewis and Kretschmer [18] proposed an approach in which they proved that one can use polyphase codes instead of using bi-phase codes, as the polyphase codes are also referred to compress the pulse of the given signal waveform to attain enhanced PSR and can be used to avoid the security problem. This method is very accurate to cause P1 and P2 polyphase codes. These polyphase codes are generated from the step estimation by the use of a modulation technique called linear frequency modulated waveform technique (LFMWT). Such polyphase codes have an additional tolerance to the restriction of the receiver side bandwidth

Sindhura et al. [19] proposed a model, based on a wavelet, in this paper the authors compare the signal to noise ratio enhancement for Lower Atmospheric Signals. This approach is used to calculate the wind performance in the atmospheric boundary layer (ABL) and minor troposphere. SakhaWat et al. [20] presented an approach in which the author gets the information of GPS signals and can be used to present a novel use to remote-sensing since they are capable of providing valuable information concerning the reflecting face. However, this technique puts much attention towards image arrangement that too of fixed (static) targets only, hence cannot have an optimal use in moving target detection. Syed and Venay [21] projected a technique in which the main focus was to improve signal loss. Here they use p1, p3 codes, and hyperbolic frequency modulation (HFM) polyphase codes are used to increase the signal-to-noise ratio of the acknowledged signal. However, this approach has an adverse effect of delay, therefore not suitable to detect the multiple moving targets. Singh et al. [22,23] projected two different coding techniques to minimize the noise. No doubt the authors improved the count of the windows which can enhance the probability of target detection. However, these methods are limited to finding stationary and slow-moving objects only, since the period of the designed code vector is not as much of which minimizes the merit factor (MF) of the received echo signal and has noise in the region of the zero Doppler. In [24,25] two coding techniques have been presented to enhance the target detection in Doppler tolerant radar. Though, both techniques exploit the mathematical involvedness and maximize the delay parameter, which decreases the probability of target detection and could not be the optimum method to find -moving targets. Alotaibi [26] presented a technique of target detection using linear block coding, in which the author generates the code using well-known (6,3) block code and then inserts the odd and even parity to change the present position of the code word to achieve the result. However the presented approach uses more number of gates to complement the existing code to increase the length of the codeword which increases the hardware and delay therefore, may not be optimum to detect multi-moving targets in Doppler tolerant radar. The digital code techniques [24,25,26] are only concentrating on the Doppler but not on the auto and cross-correlation properties of the sequences which are considered to be important to detect the desired target in a multi-target environment. In [27] the authors designed the digital sequences with optimal properties to detect the static and moving targets. But the side noise in this method is more when compared to the presented approach (refer to Section 4 as comparative analysis of the existing approach and the proposed approach is depicted in the same section).

In this paper, an approach is being presented to optimize the interference to detect static and moving objects. The presented approach helps to detect multiple moving targets efficiently as the sequences obtained are tested for auto and cross-correlation along with the Doppler tolerant frequencies.

## 3. Proposed Approach

Initially, a concatenation series of decimal numbers having the equal number of zeros and ones when represented in hex code (i.e., from 0 to 15) are considered. This series is termed binary hex equal-weighted codes (BHEWC) and can be represented as

(1) $$I_{CW}=\prod_{p=1}^{4}3p,\;1\leq p\leq 4$$

where ‘$I_{CW}$’ is concatenation series of BHEWC.

The concatenation series then will be 3, 6, 9, and 12; the same series in binary hex value can be represented as ‘0011011010011100’. The MSB bit of the initially designed code is zero and it doesn’t satisfy the cyclic division rule (as the cyclic division process is used to generate the codes) so the hex value of the first decimal number is given one bit left shift (i.e., 0011 = 1001), and thus the concatenation series can now be represented as 1001011010011100. The remaining decimal numbers having equal weight (i.e., missed numbers in Equation (1)) can be formulated as

(2) $$M_{B1}=\frac{I_{TS}+L_{TS}}{I_{TS}}$$

M_B2_ = 2 M_B1_ (3)

‘$I_{TS}$’ is the first decimal value of ‘$I_{CW}$’ and

‘$L_{TS}$’ is the last decimal value of ‘$I_{CW}$’

Now from Equations (2) and (3) M_B1_ = $\frac{3+12}{3}$ = 5 (Binary hex value 0101) and M_B2_ = 2 × 5 = 10 (Binary hex value 1010) are the missing decimal numbers of ‘$I_{CW}$’. Later cyclic redundancy method is used to improve the target detection and minimize the noise near-zero Doppler, in which the generator codeword can be represented by ‘$G_{CW}$’ and the Radar codeword generation using cyclic process can be characterized as

(4) $${\left(R_{CW}\right)}_{CP}=\frac{I_{CWp_{i}}}{G_{CW}},\;i=1,\;2$$

where ‘$I_{CWp_{i}}$’ is the code after appending ‘$p_{1}$(q)’ or ‘$p_{2}$(q)’ zeros

(5) $$p_{1}(q)=p_{2}(q)=-1$$

where r is the hex code length of M_B1_ or M_B2_ (as they are of the same length).

Therefore, the length is 4 bits for both the cases, thus 4 − 1 = 3 zeros need to append to ‘$I_{CW}$’ to get $I_{CWi}$ codes that are employed in the cyclic process to get the initial radar codes to test the target. In this approach, modulo 2 additions are employed to generate the codeword. However, the code sequence that is exploited to generate the initial codeword to detect the targets in Doppler tolerant radar can be represented as

(6) $$R_{1CW}=r_{cp1}\;⊕\;I_{CWp_{1}}$$

(7) $$R_{2CW}=r_{cp2}\;⊕\;I_{CWp_{2}}$$

where ‘$r_{cp1}$’, ‘$r_{cp2}$’ are the remainders when ‘M_B1_’ and ‘M_B2_’ are the divisors of Equation (4) respectively, However as the MSB (most significant bit) of M_B1_ is ‘0’, therefore to satisfy the cyclic process one bit left shift has given to ‘M_B1_’. Hence the generated divisor can be represented as ‘1100’ and the two initial code words that are used to generate the final radar target detection code words can be represented in Table 1 and Table 2 respectively.

The final codeword matrix is formulated and can be represented in Table 3 whose first row is ‘$R_{1CW}$’and first column is ‘$R_{2CW}$’

The remaining elements of the matrices are formulated by modulo two operations of row and Column and can be represented as

(8) $$r_{11}=b_{1},\;r_{12}=b_{2},\ldots \ldots r_{1n}=b_{n}$$

(9) $$r_{21}=\;c_{2},\;r_{31}=c_{3},\ldots \ldots r_{n1}=c_{n}$$

(10) $$r_{12}=b_{2}\;⊕\;c_{2},\;r_{n2}=b_{2}\;⊕\;c_{n},\;r_{nn}=b_{n}\;⊕\;c_{n}$$

**Figure 1:**
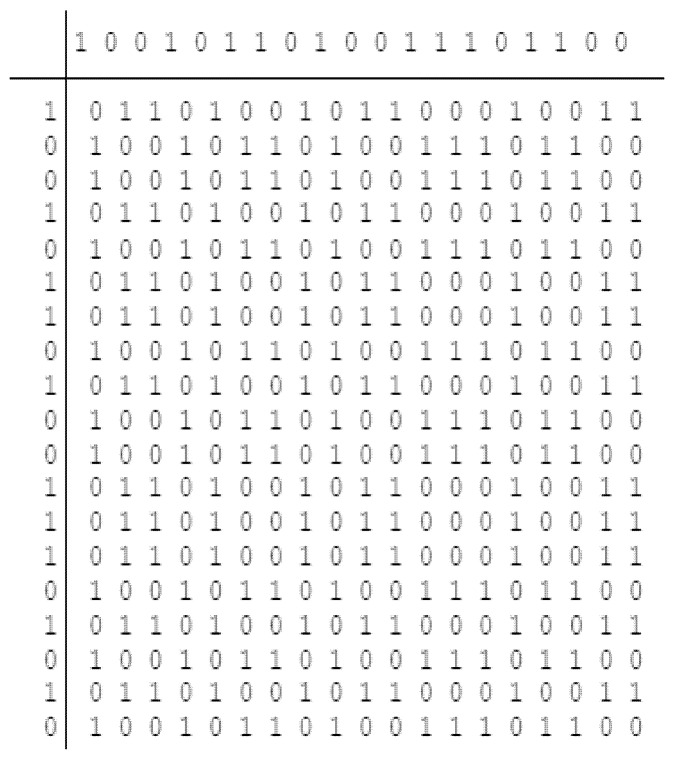
Binary code matrix.

The matrix formed is represented in Figure 1. From the figure, 19 × 19 matrix without considering the initial codes $R_{1CW}$ (first row) and $R_{2CW}$ (first column) is obtained.

To design the codes which are Doppler tolerant and with optimal autocorrelation and cross-correlation properties can be generated by performing various mathematical operations on the matrix such as

a. Concatenation of rows,
b. complement operation is performed on even terms,
c. if continuous series of three 1’s and 0’s occur in the sequence then the middle term is complemented and

The bits which are at the position of the sum of the power of 2 are complemented (i.e., multi-criteria creation of digital codes to test the target).

**Figure 2:**
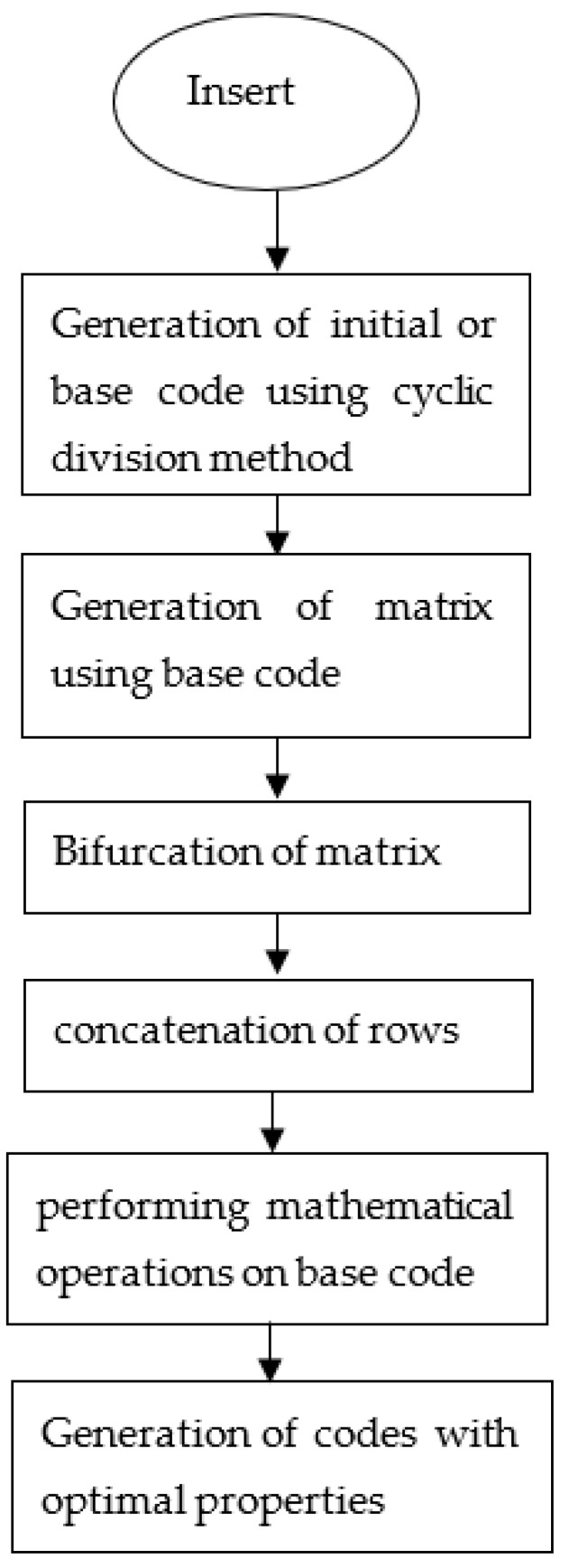
Flow diagram of the proposed approach.

Note that one need not perform all the operations to obtain the sequence with good properties. The sequence obtained after performing each operation is tested and the sequence with the best properties (maybe after performing one operation, two operations, all the operations, or no operation) is selected. Figure 2 shows the flow diagram of the proposed approach. The codes of different lengths (here 19-bit, 95-bit, and 190-bit codes) are generated from the above operations. The ambiguity, auto-correlation, and cross-correlation which are the optimal characteristic properties of static and moving target detection in a multi-target environment are observed using the mat-lab tool. For testing the sequence for auto and cross-correlation properties the 0’s of the sequence is replaced by −1 to obtain the actual length of the sequence and for the ambiguity function, this change is not necessary. The simulation parameters are shown in Table 4.

**Figure 3:**
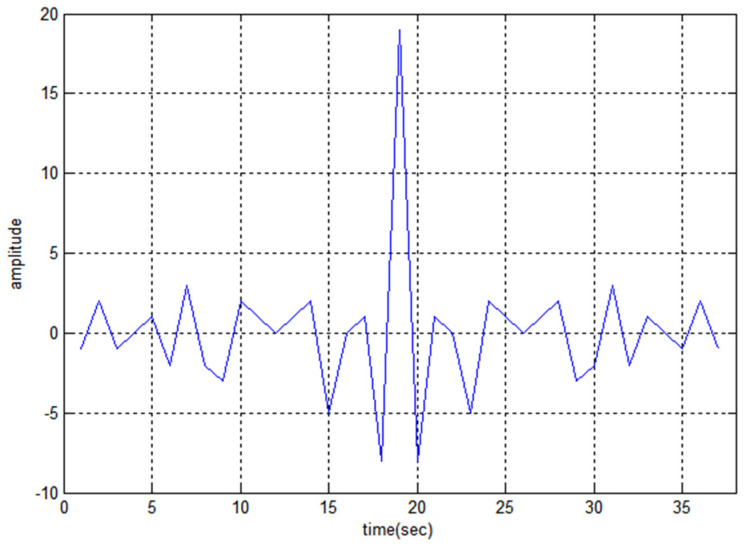
Auto-Correlation property of$\;R_{2CW}$.

**Figure 4:**
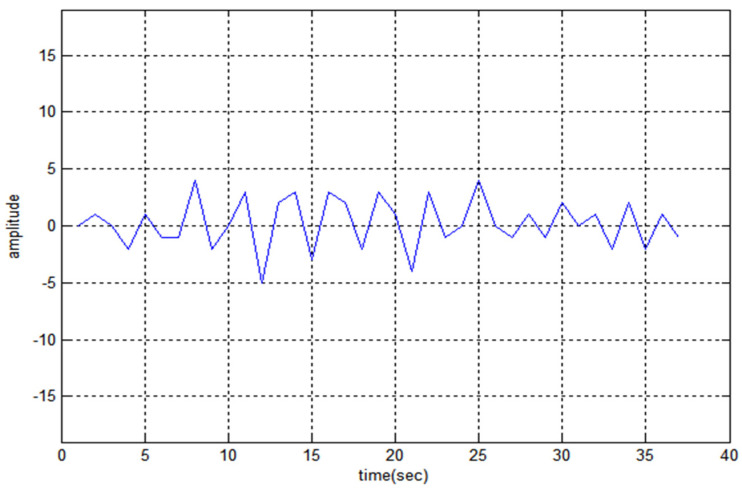
Cross-Correlation property of$\;R_{2CW}$.

**Figure 5:**
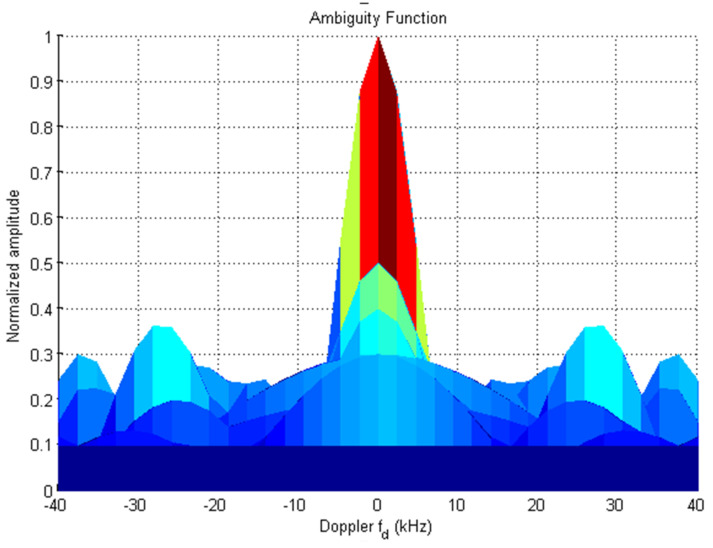
Ambiguity-Function of$\;R_{2CW}$.

The 19-bit code $R_{1CW}$ and $R_{2CW}$ are directly tested (without performing any mathematical operation) for the above properties and $R_{2CW}$ shows better response, unlike$\;R_{1CW}$. Figure 3, Figure 4 and Figure 5 depict the auto-correlation, cross-correlation, and Doppler tolerance of the code$\;R_{2CW}$.

From the graph, the side noise amplitude has the amplitude of 2 and the reduction ratio of peak to side-lobe $S_{R}$ in dB can be calculated as [27].

(11) $$S_{R}=20log_{10}\;\left(\frac{amplitude\;of\;side\;noise\;which\;is\;maximum\;}{amplitude\;of\;main\;lobe}\right)=20log_{10}\;\left(\frac{\;2}{19}\right)=-19.56\;(\mathrm{approx})\;\mathrm{dB}$$

From Figure 3 the maximum lobe for cross-correlation is 4, and Figure 4 shows the ambiguity function of $\;R_{2CW}$ code. It is observed that there are no clear windows below 0.2 (normalized amplitude which is considered as standard threshold value), the clear windows are present above 0.3 normalized value.

## 4. Generation of 95-Bit and 190-Bit Sequence

**Figure 6:**
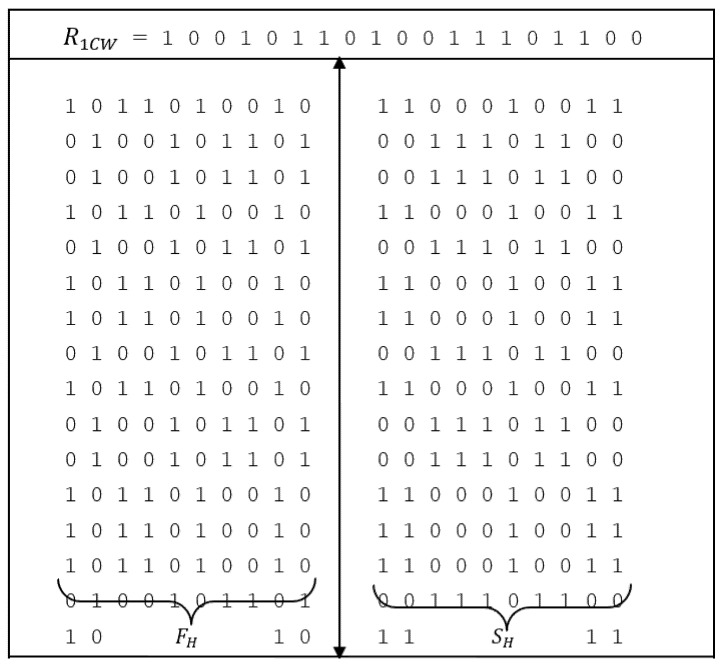
Generation of 190-bit code.

**Figure 7:**
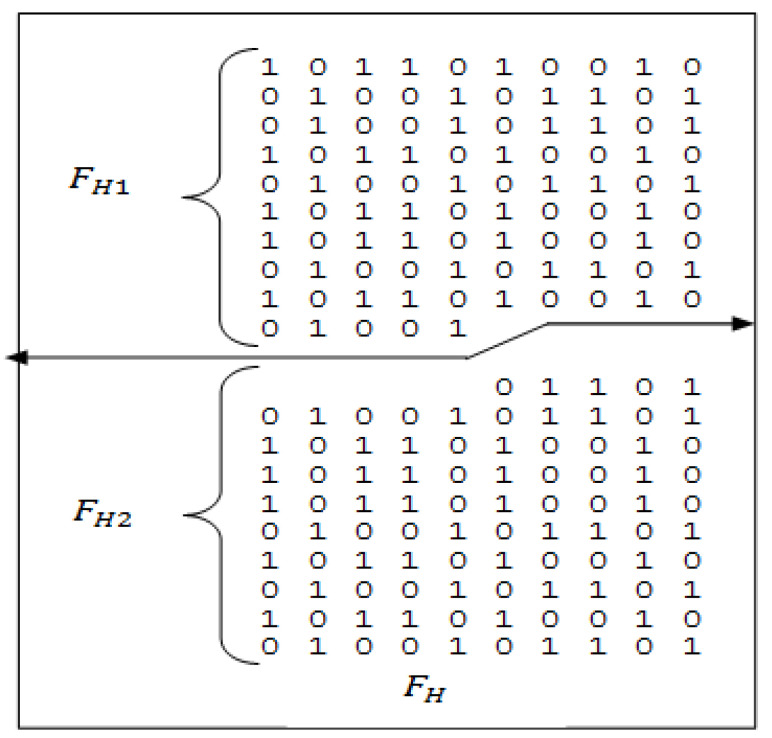
Generation of 95-bit code.

The 190-bit and 95-bit code is generated from the matrix of Figure 1. Initially, the first row (${\;R}_{1CW})$ is eliminated to get the matrix of 19 × 20, however one can eliminate the first column ($R_{2CW}$) to get 20 × 19 and test the codes. The codes of length 190-bit and 95-bit are generated as represented in Figure 6 and Figure 7 respectively. The arrow in Figure 6 represents the matrix division vertically into two parts termed the first half ($F_{H}$) and the second half ($S_{H}$) respectively to obtain the desired length of codes.

The sequence of 190-bit is obtained by concatenation of $F_{H}$ (refer to Figure 5). The mathematical operations performed on this 190-bit sequence respectively are (i) complementing even terms, (ii) complementing the middle bit if three continuous 0’s or 1’s occurs, and lastly (iii) complementing the bits present at the power of 2 positions. After performing all these operations the final code (F1) obtained can be represented as

F1 = 1 1 1 1 1 0 0 0 0 1 0 1 0 1 0 0 1 0 1 0 1 0 0 1 0 1 1 0 1 0 1 1 1 0 1 0 0 1 1 0 1 0 1 0 1 0 0 1 0 1 0 1 0 1 0 1 1 0 1 0 1 0 0 0 0 1 1 0 1 0 1 0 1 0 1 0 0 1 0 0 1 0 1 0 1 0 0 1 0 1 0 1 0 1 0 1 1 0 1 0 1 0 0 1 0 1 1 0 1 0 1 0 1 0 1 0 1 0 1 0 1 0 0 1 0 1 1 1 1 0 1 0 0 1 0 1 1 0 1 0 1 0 1 0 1 0 0 1 0 1 0 1 0 1 0 1 0 1 0 1 0 1 0 1 0 1 1 0 1 0 1 0 1 0 1 0 0 1 0 1 0 1 0 1 0 1 1 0 1 0.

The code obtained by concatenation of $F_{H}$ performs better than the codes obtained by concatenation of $S_{H}$.

The sequence of 95-bit length is generated by horizontally dividing the first half ($F_{H}$) of the matrix equally as represented in Figure 7. Let the two equal parts be $F_{H1}$ and $F_{H2}$ respectively.

**Figure 8:**
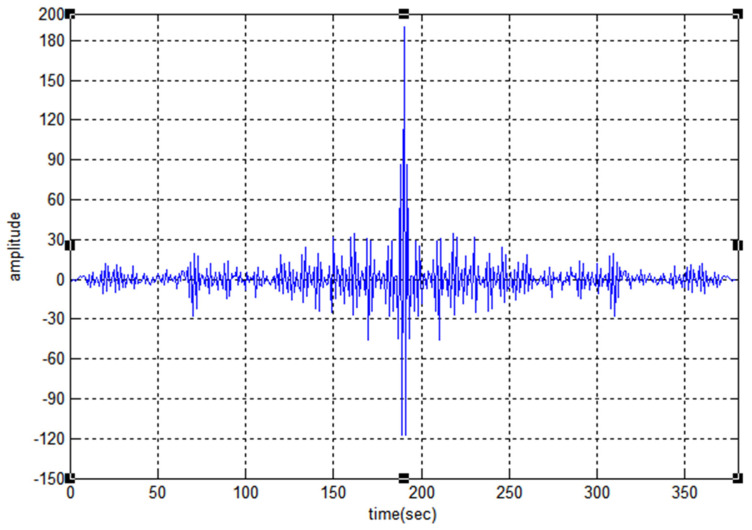
Auto-Correlation property of F1.

**Figure 9:**
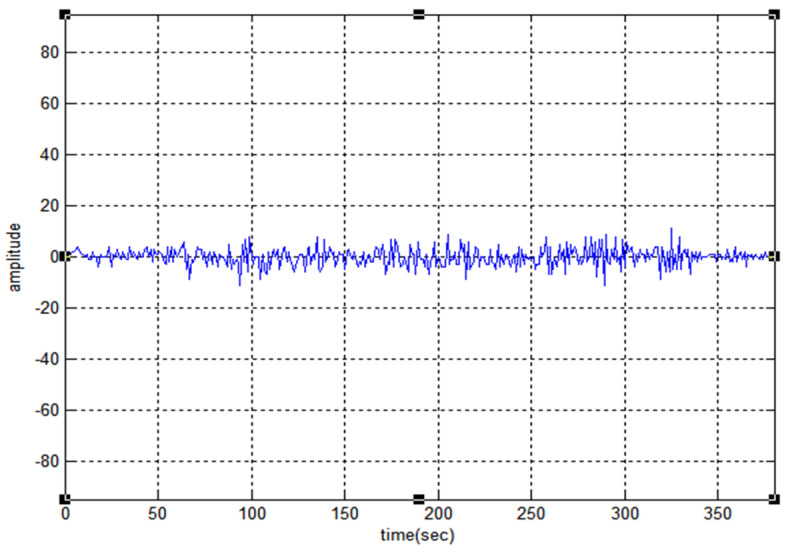
Cross-Correlation property of F1.

**Figure 10:**
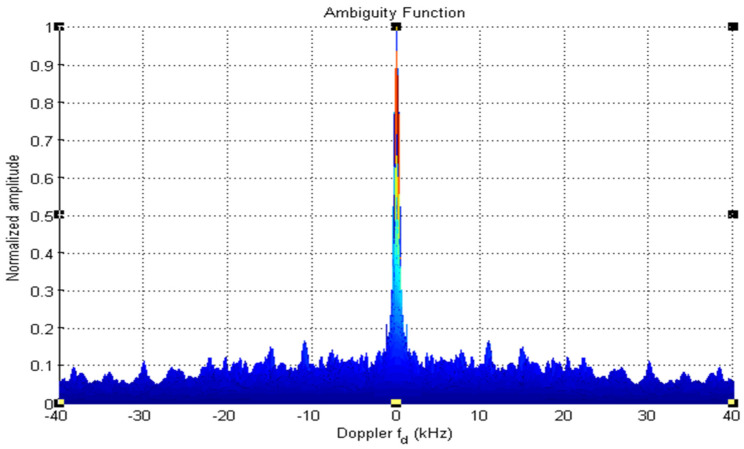
Ambiguity-Function of F1.

Figure 8 shows the auto-correlation property of code F1 and the maximum noise lobe is 30 the $S_{R}$ value is −16.03. Figure 9 and Figure 10 represent the cross-correlation and Doppler tolerance of sequence F1. The noise in Figure 10 is below 0.2 (normalized amplitude) and after 24 kHz frequency, the noise is almost zero.

From Figure 7 the generation of 95-bits is shown (concatenation of$\;F_{H1}$ and$\;F_{H2}$), as the concatenation of $F_{H2}$ generates better properties than$\;F_{H1}$. The 95-bit sequence (F2) tested in this paper is obtained by complementing the even terms of the code generated by concatenation of$\;F_{H2}$.

(F2) = 0 0 1 1 1 0 1 1 1 0 0 0 0 1 1 0 0 0 0 0 0 1 0 1 1 0 0 1 1 1 1 0 1 0 0 1 1 0 1 0 0 0 1 1 1 0 0 0 1 1 1 1 0 1 1 1 1 0 1 1 0 0 0 0 1 0 1 1 1 1 0 0 0 0 1 0 1 1 1 1 1 1 0 0 0 1 1 1 0 0 0 0 0 0 1

**Figure 11:**
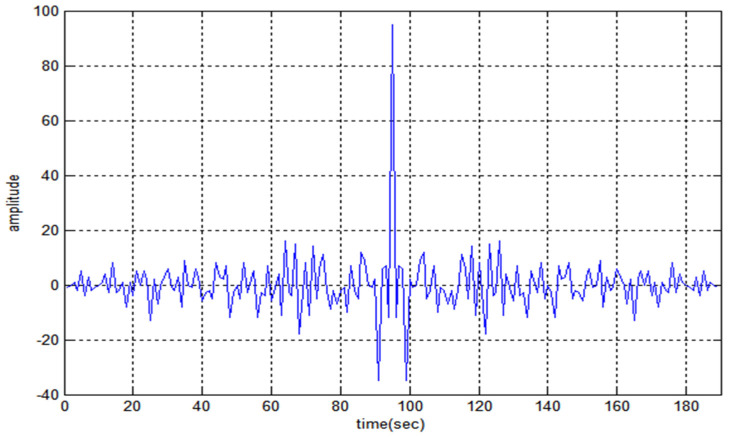
Auto-Correlation property of F2.

**Figure 12:**
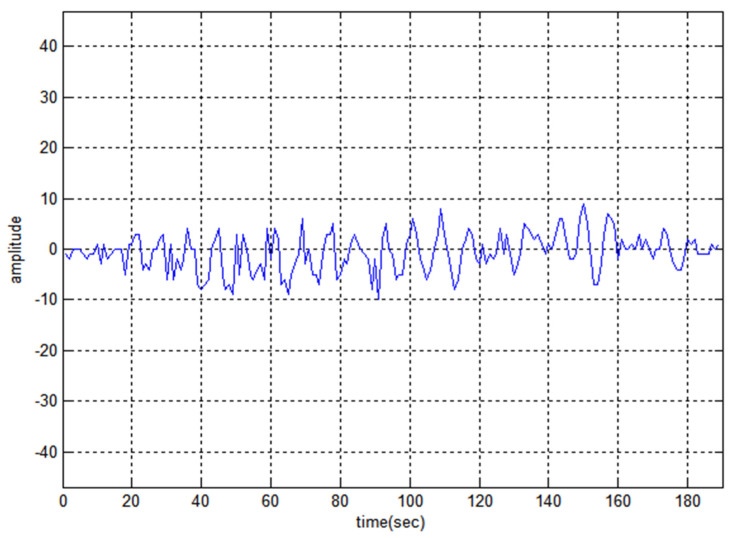
Cross-Correlation property of F2.

**Figure 13:**
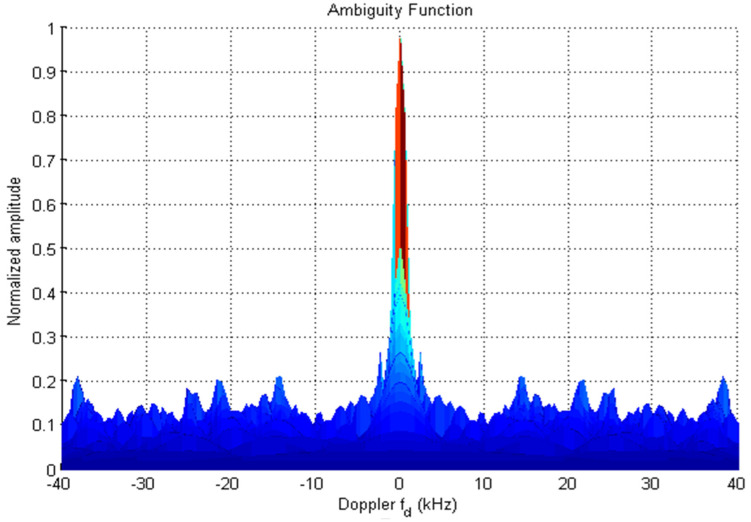
Ambiguity-Function of F2.

Figure 11, Figure 12 and Figure 13 depict the auto-correlation, cross-correlation, and ambiguity of sequence F2. The value of $S_{R}$ for F2 is −15.48. In Figure 13 the noise amplitude is 0.2 at three frequencies and less than it for other frequencies with clear windows at 3–14 kHz, 16–21 kHz, and 24–38 kHz.

**Figure 14:**
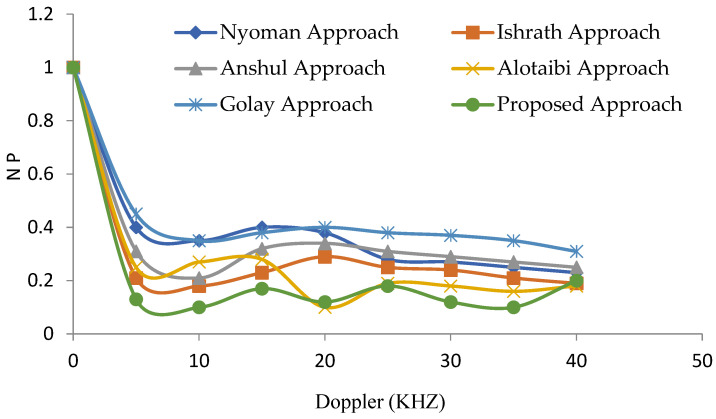
Noise peaks Vs Doppler.

Figure 14 represents the noise peak (NP) versus the Doppler graph. It presents the comparative analysis of various existing approaches [26,27] with the proposed approach and it has been observed from the figure that the presented approach shows better performance with minimal side noise peaks when compared with the existing approaches as the presented approach obtained the digital codes with clear windows to detect the desired target in a multi-target environment.

Table 5 represents the length of the sequence and parameters achieved in terms of peak to side-lobe noise amplitude $S_{R}$ in dB, cross-correlation noise amplitude, and clear windows at different Doppler’s.

## 5. Discussion

The designed sequences can be used to detect the static as well as moving targets in multi-target ambiance. From the simulated results it has been shown that the auto-correlation, cross-correlation, and Doppler tolerance properties of the designed codes are better in comparison with the existing approaches [28,29,30], as they are of a limited length and used for only static target detection. The Auto, cross-correlation and Doppler tolerance properties of the proposed approach adhere that the presented approach finds an extensive use to find the desired target in presence of Doppler. The proposed approach is simple as it generates the digital code sequences with different lengths. There is no limitation to the length of the sequences as one can design the codes of any length using any mathematical operations. The codes are cost-effective as they are obtained without employing any extra costly hardware components. As the presented digital codes are achieved from a single basic code i.e., binary hex equal-weighted code, by revising these codes using fundamental laws of mathematics and communication code theory, the stage of range gate in the phase of target detection is reduced which decreases the cost and hardware and minimizes the delay. Therefore can be employed to see the fast and multiple moving targets above MAC-3 i.e., latest generation combatant crafts by opting for a suitable code length with optimal properties to reduce all the noise amplitude peaks lower than 0.2 dB (normalized amplitude) which is the standard threshold value [23].

## 6. Conclusions

Radar systems utilize two or more antennas to detect the target in a multi-target environment. Hence, the interference of the sequence with itself and with other sequences should be optimal for static target detection and the sequences should be Doppler tolerant for the detection of moving targets. The clear windows obtained in Figure 9 and Figure 12 are used to detect the moving targets at desire Doppler. Hence, the codes obtained in this paper can be used to detect the static and moving targets accurately with optimal interference and improvised range and resolution.

## Figures and Tables

**Table 1:** Binary representation of $R_{1CW}$.

$R_{1CW}$	*b*_1_	*b*_2_	*b*_3_																*b_n_*
	1	0	0	1	0	1	1	0	1	0	0	1	1	1	0	1	1	0	0

*b*_1_, *b*_2_, *b*_3_,……, *b_n_* bit positions of code $R_{1CW}$.

**Table 2:** Binary representation of $R_{2CW}$.

$R_{2CW}$	*c*_1_	*c*_2_	*c*_3_																*c_n_*
	1	0	0	1	0	1	1	0	1	0	0	1	1	1	0	1	0	1	0

*c*_1_, *c*_2_, *c*_3_,……, *c_n_* bit positions of code $R_{2CW}$.

**Table 3:** Final codewords using matrices.

$r_{11}$	$r_{12}$	-	-	-	-	-	-	-	-	-	-	-	-	-	-	-	$r_{1n}$
	-																-
	-																-
	-																-
$r_{n1}$	$r_{n2}$	-	-	-	-	-	-	-	-	-	-	-	-	-	-	-	$r_{nn}$

**Table 4:** Simulation Parameters.

Transmitter	
Power of pulse	470 kW
Frequency	2800 MHz to 3100 MHz
RF duty cycle	Max 0.004
Width of the pulse	1.55 μs and 4.50 μs
Receiver	
Intermediate frequency	55.5 MHz
Range (dynamic)	95 dB
noise power of the system	–112 dBm
Target (in σ meter square)	
Man	0.14 to 1.05
Aircraft C-54	10 to 1000
A free electron	8 × 10^−30^
Birds	10^−3^

**Table 5:** Parameters of the obtained sequences.

Sequence Length	*S_R_* in dB	Max. Noise Amplitude (Cross-Correlation)	Clear Windows of Ambiguity Figure
19	−19.56	4	above 0.3 normalized amplitude
95	−15.48	8	3–14 kHz, 16–21 kHz, and 24–38 kHz.
190	−16.03	9	0–40 Hz

## Data Availability

Not applicable.
